# Conventional three-port laparoscopic appendectomy versus transumbilical and suprapubic single-incision laparoscopic appendectomy using only conventional laparoscopic instruments

**DOI:** 10.1007/s00423-022-02683-6

**Published:** 2022-09-20

**Authors:** Shaohan Wu, Yiyu Shen, Jing Wang, Jinquan Wei, Xujian Chen

**Affiliations:** 1 Department of General Surgery, The Second Affiliated Hospital of Jiaxing University, Huancheng north road, Jiaxing, 314000 NO. 1518Zhejiang China; 2 Department of General Surgery, Feixian People’s Hospital, Linyi, 273400 Shandong China

**Keywords:** Appendectomy, Laparoscopy, Umbilicus, Pubic symphysis, Single-incision

## Abstract

**Purpose:**

Single-incision laparoscopic appendectomy (SILA) is usually performed using single-port instruments, which may restrict its development and application. This study explored the performance of transumbilical SILA (TSILA) and suprapubic SILA (SSILA) using only conventional laparoscopic instruments and compared them with conventional three-hole/port laparoscopic appendectomy (CLA).

**Methods:**

This retrospective study included 174 patients who underwent CLA, TSILA, or SSILA for acute appendicitis at our hospital between June 2019 and July 2021. Demographic data and clinical outcomes were compared among the three groups.

**Results:**

Compared with CLA, TSILA was associated with significant reductions in postoperative pain, length of hospital stay, and hospital cost, while SSILA was associated with significant reductions in length of hospital stay and hospital cost (all *P* < 0.05). Significantly more patients in the two SILA groups were cosmetically satisfied than those in the CLA group (all *P* < 0.05). However, compared with CLA, SSILA required a significantly longer operative time (65.3 ± 24.1 vs 56.5 ± 20.9, *P* = 0.039). Besides, compared with TSILA, SSILA showed significantly higher postoperative pain score (2 ± 2 vs 3 ± 2, *P* = 0.006). Mild incisional or intraabdominal infections were noticed in 2 (3.0%) patients in the CLA group, 3 (5.1%) in the TSILA group, and 3 (6.3%) in the SSILA group (*P* = 0.69).

**Conclusion:**

SILA performed with only conventional laparoscopic instruments was associated with reduced hospital stay and cost and higher cosmetic satisfaction in comparison to CLA. However, it is technically demanding and may increase operative time.

## Introduction

Acute appendicitis is one of the most common abdominal diseases in general surgery [1]. Laparoscopic appendectomy was first reported in 1983 [2]. Subsequently, it gradually replaced open appendectomy for its advantages such as less surgical site infection, faster postoperative recovery, and better cosmetic effects [1, 3]. Conventional three-hole/port laparoscopic appendectomy (CLA) has been the “gold standard” for the treatment of acute appendicitis. Single-incision laparoscopic appendectomy (SILA), such as transumbilical single-incision laparoscopic appendectomy (TSILA) and suprapubic single-incision laparoscopic appendectomy (SSILA), is thought to further reduce surgical trauma, pain, and scars [4–6]. However, there are still many controversial results regarding single-incision laparoscopic SILA compared to CLA [7–9].

The laparoscopic technique was initially developed for a single disease, and now it has evolved into a multidisciplinary and multi-path surgical technique. Furthermore, robotic laparoscopy, natural orifice translumenal endoscopic surgery, and single-port laparoscopic surgery have become important directions for the development of minimally invasive technology in the twenty-first century [7, 10]. SILA is mainly performed using self-made or commercial single-port instruments [7, 11–15]. However, the instruments or technical requirements for these advanced surgical techniques are often not available in underdeveloped regions or countries, which restrict the development and application of SILA. Then, some surgeons have tried to perform TSILA with conventional laparoscopic equipment and have achieved reliable results [7, 16, 17].

In the opinion of some patients and surgeons, there are some other concerns about TSILA. For example, it may lead to umbilical deformation, incisional hernias, and incision infections. Also, in Chinese and East Asian cultures, the umbilicus is believed to be associated with health and longevity and should avoid any harm or injury. The undesirable cosmetic effect of TSILA also discourages some patients. Therefore, SSILA may provide an attractive alternative to TSILA for these patients. After that, some surgeons have tried SSILA with self-made or commercial single-port laparoscopic equipment. They demonstrated the reliable safety and feasibility of SSILA [5, 6, 18]. However, SSILA without single-port instruments has not yet been reported.

The present study aimed to compare the perioperative outcomes between CLA, TSILA, and SSILA at a single institution, which were all performed with conventional laparoscopic instruments. The surgical techniques were also summarized.

## Materials and methods

### Patients

This retrospective study analyzed patients who were surgically treated for acute appendicitis between June 2019 and July 2021 at our hospital by a single experienced surgeon (Chen XJ) with more than 10 years of experience with hepatobiliary and gastrointestinal surgery, such as CLA, and with more than 3 years of experience with SILA. The decision of surgical procedures of CLA, TSILA, or SSILA was made by the surgeon and the patients, considering each patient’s individual condition, surgical risks, and cosmetic requirement. Sex, age, body mass index (BMI), preoperative disease course, operative time, intraoperative blood loss, postoperative pain degree, C-reactive protein (CRP) level on the first day after surgery, surgical complications, length of hospital stay, hospital cost, and degree of satisfaction with the cosmetic results were collected. The operative time was recorded from the first skin incision to the final skin suture. The study protocol was approved by the ethical committee of our hospital.

### Inclusion and exclusion criteria

All patients met all of the following criteria: disease course less than 72 h, no severe diseases in other organs, acute appendicitis confirmed with postoperative pathological results, age between 13 and 75 years, and complete medical records. AA was preoperatively diagnosed according to medical history, symptoms, signs, inflammatory biomarkers, and imaging examinations, mainly including computed tomography or ultrasonography. Patients who met one of the following criteria were excluded: incomplete follow-up data, height < 1.5 m, BMI > 28, serious cardiopulmonary diseases or other serious diseases requiring long-term treatment, coagulation dysfunction, serious organ dysfunction or other surgical contraindications, pregnancy.

### Surgical instruments

We used conventional laparoscopic instruments (Stryker laparoscopic system, USA; OLYMPUS laparoscopic system, Japan) equipped with a 10-mm 30° laparoscope, metal trocars, bipolar electrocautery, monopolar electrocautery, and other supporting laparoscopic instruments. A ligation device (BD13008, Shandong Boda Medical Supplies, China) was used to ligate the appendix stump.

### Surgical technique

**Fig. 1:**
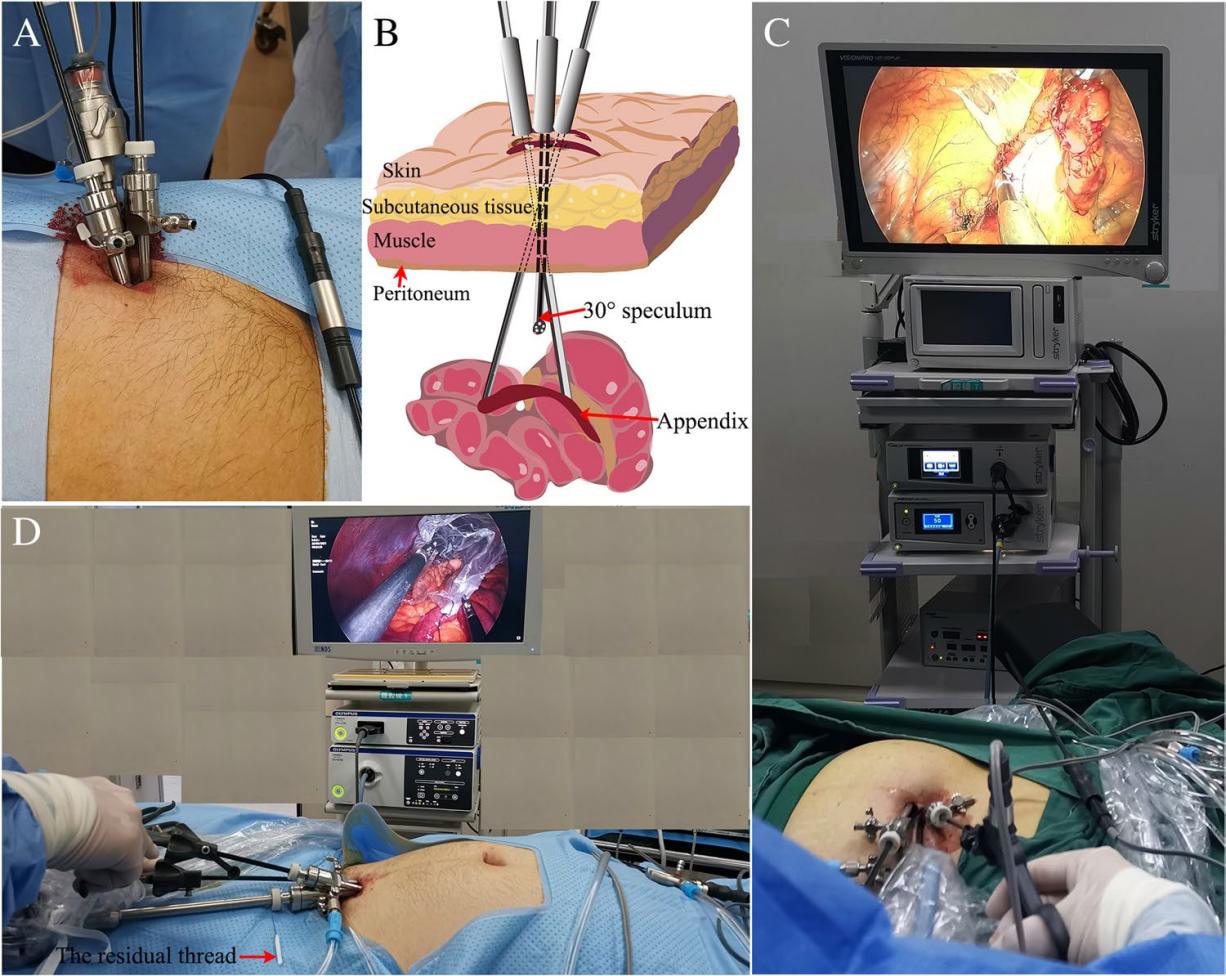
The TSILA and SSILA techniques were performed using only conventional laparoscopic instruments and without the aid of any single-port instruments. The scope of the operation and the visibility were both clear. **A** and **B** Three trocars were brought together and respectively inserted into the abdominal wall and the peritoneum layer via a 2-cm skin incision around the umbilicus. **C** TSILA was performed and used to remove the appendix with conventional laparoscopic instruments through a 2-cm incision around the umbilicus. **D** SSILA was performed via a 2-cm incision. A 2-cm incision was made approximately 3 cm above the pubic symphysis

For TSILA, the operating table is modified in the Trendelenburg position. A 10-mm arc incision is incised around the left edge of the umbilicus to establish pneumoperitoneum of 12 mmHg. Then, a 10-mm trocar is vertically inserted into the abdominal cavity. The appendix and the abdominal cavity are explored for the first time with a 30° laparoscope. If there is no gangrene or perforation of the appendix base or difficulty in exposing the appendix, laparotomy, or three-hole LA is not performed. The incision is then extended to 15 mm. Subsequently, a 5-mm trocar is obliquely inserted into the abdominal cavity to determine the ileocecal area and the location of the appendix for the second time. If a two-hole LA is not required, the original incision is extended to 20 mm. Another 5-mm trocar is obliquely inserted into the abdominal cavity along a different path. This creates a tissue space between the three metal trocars in the muscular layer to prevent air leakage (Fig. 1A and B).

**Fig. 2:**
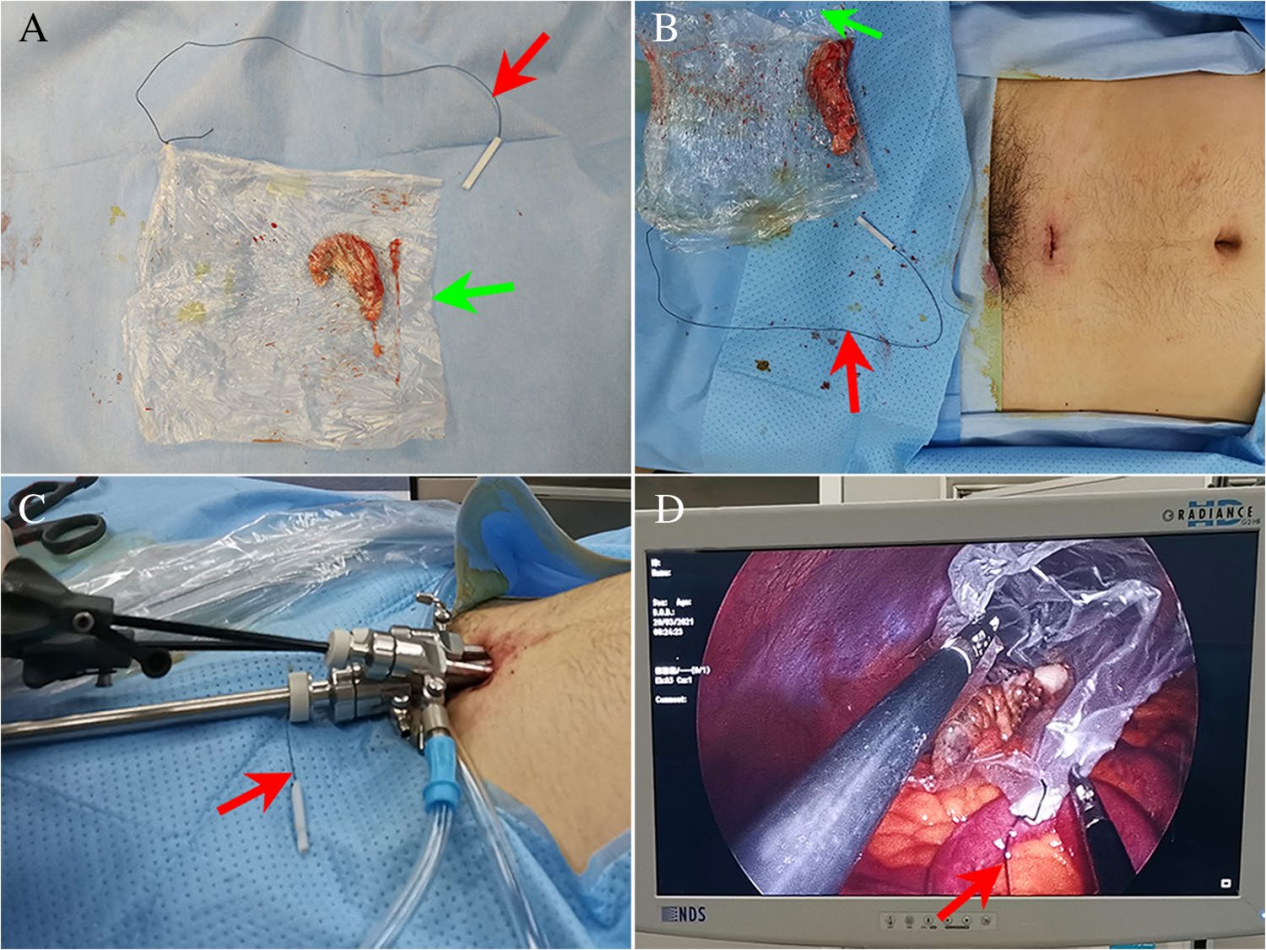
The residual thread of the ligation instruments and a plastic bag were used to make a retrieval bag. Red arrows point to the residual thread. Green arrows point to the plastic bag. **A** and **B** The appendix was removed from the abdominal cavity using a self-made retrieval bag. **C** The distal end of the remnant thread was outside the trocar. **D** The end of the residual thread was tied to the plastic bag in the abdominal cavity

The three trocars are arranged in an arc and present an “operating triangle” inside the abdominal cavity to improve operative exposure, reduce the blind area of the operation, and minimize the inconvenience of single-incision operations. The assistant holds a laparoscope and stands to the right rear of the surgeon and on the left side of the patient’s left shoulder. All laparoscopic instruments are pointed from the umbilicus to the appendix (Fig. 1C). Bipolar electrocautery and monopolar electrocautery are used to deal with the mesoappendix and the stump of the appendix. A ligation device is used to ligate the appendix stump. The residual appendix stump was closed with two knots. And the resected appendix was also closed with a knot. The inflamed appendix is placed in a self-made plastic bag and removed from the abdomen through an enlarged incision. The edge of the bag is tied to one conventional suture or the residual thread of the ligation device. The bag is introduced through a 10-mm trocar. The distal end of the thread is placed outside the 10-mm trocar to easily pull out the appendix and avoid wound infection. The other end of the residual thread is tied to the bag (Fig. 2). The inflamed appendix is removed from the abdomen through an enlarged periumbilical incision. The length of the incision remains unchanged. In the end, we used a cosmetic method of suturing for the incision closing.

For SSILA, a 15-mm transverse incision is made approximately 3 cm above the pubic symphysis at the linea alba (Fig. 1D). Subsequently, the subcutaneous tissue and the linea alba are cut longitudinally. Then, the abdominal wall is dissected layer-by-layer using the open access technique, namely the direct fascial puncture technique [16, 19]. Two towel forceps are used to lift the abdominal wall at the only incision from both sides. Then, a 10-mm trocar is inserted into the abdominal cavity to establish pneumoperitoneum of 12 mmHg. Two 5-mm trocars are inserted into the abdominal cavity along different paths. The method of exploring the abdominal cavity and extending the incision is the same as that used for TSILA. The assistant stands between the patient’s legs, and the surgeon stands on the left side of the patient’s left leg. The operating instruments are pointed from the pubic symphysis to the appendix (Fig. 1D). The methods for dissection, ligation, excision, and removal of the mesoappendix and appendix in SSILA are the same as that in TSILA in this text and that in reported SSILA using single-port instruments [5, 20].

CLA is performed using the modified three-hole LA with one 10-mm trocar and two 5-mm trocars. The methods for ligation and removal of the appendix in CLA as well as the cosmetic method of suturing for the incision closing are similar to TSILA in this text.

### Postoperative evaluation

The visual analog scale was used to evaluate postoperative pain. The pain severity was graded as no pain (0 point), mild pain (1 to 3 points), moderate pain (4 to 6 points), and severe pain (7 to 10 points).

Satisfaction survey questionnaires were used to assess patients’ degree of satisfaction with their cosmetic results, including satisfied, acceptable, and unsatisfied. Higher scores indicated more favorable cosmetic outcomes.

### Statistical analysis

All data were analyzed using the SPSS 23.0 statistical software package (SPSS Inc., Chicago, IL, USA). The numerical data that followed a normal distribution were expressed as mean ± standard deviation and were analyzed using the *t*-test or the ANOVA analysis. The numerical data that followed a non-normal distribution were expressed as median ± interquartile range and were analyzed using the Mann–Whitney *U* test or the Kruskal–Wallis test. The chi-square test was used to analyze the categorical data. *P* values less than 0.05 were considered statistically significant.

## Results

The final analysis included 174 patients, with 59 in the TSILA group, 48 in the SSILA group, and 67 in the TLA group. There were no significant differences among the three groups in terms of sex, age, weight, BMI, preoperative disease course, intraoperative blood loss, and CRP level on the first day after surgery (Tables 1 and 2).

**Table 1:** Patient demographic variables

Variables	CLA group (*N* = 67)	TSILA group (*N* = 59)	SSILA group (*N* = 48)	*P*_*1*_ value	*P*_*2*_ value	*P*_*3*_ value
Age (year)	41.99 ± 18.18	37.86 ± 19.77	39.00 ± 16.47	0.209	0.389	0.750
Sex (male/female, *N*)	32/35	23/36	26/22	0.410	0.451	0.618
Height (m)	1.65 ± 0.07	1.66 ± 0.08	1.69 ± 0.09	0.519	**0.038**	0.153
Weight (kg)	62.48 ± 10.58	64.15 ± 10.23	63.77 ± 12.39	0.395	0.535	0.858
Body mass index (kg/m^2^)	22.75 ± 2.64	23.08 ± 2.53	22.26 ± 2.72	0.479	0.333	0.112

*CLA*, conventional three-hole/port laparoscopic appendectomy; *TSILA*, transumbilical single-incision laparoscopic appendectomy. SSILA: suprapubic single-incision laparoscopic appendectomy. Data was shown as mean ± standard deviation. *P*_*1*_, CLA vs TSILA; *P*_*2*_, CLA vs SSILA; *P*_*3*_, TSILA vs SSILA

All the *P* values with sigificantly stastistical difference were presented in bold

**Table 2:** Patient perioperative data

Variables	CLA group (*N* = 67)	TSILA group (*N* = 59)	SSILA group (*N* = 48)	*P*_*1*_ value	*P*_*2*_ value	*P*_*3*_ value
Preoperative disease course (h)	20.10 ± 10.92	16.78 ± 10.58	18.69 ± 11.84	0.094	0.499	0.377
Operative time (min)	56.5 ± 20.9	63.0 ± 22.4	65.3 ± 24.1	0.108	**0.039**	0.592
Intraoperative blood loss (ml)	7.2 ± 4.3	5.8 ± 4.6	6.7 ± 4.1	0.088	0.546	0.325
Postoperative day 1 CRP (mg/L)	31.05 ± 22.77	33.55 ± 35.69	29.69 ± 23.84	0.618	0.798	0.480
Postoperative pain score	2 ± 3	2 ± 2	3 ± 2	**0.042**	0.498	**0.006**
Hospital stay (day)	4.4 ± 1.6	3.6 ± 1.1	3.9 ± 1.4	**0.001**	**0.032**	0.331
Hospital cost (Chinese Yuan)	9530 ± 1404	8918 ± 1921	8858 ± 1276	**0.030**	**0.025**	0.843
Satisfied with the cosmetic effect (*N*, %)	37 (55.2)	45 (76.3)	45 (93.8)	**0.013**	** < 0.001**	**0.014**

*CLA*, conventional three-hole/port laparoscopic appendectomy; *TSILA*, transumbilical single-incision laparoscopic appendectomy; *SSILA*, suprapubic single-incision laparoscopic appendectomy; *CRP*, C-reactive protein. Data are shown as mean ± standard deviation. *P*_*1*_, CLA vs TSILA; *P*_*2*_, CLA vs SSILA; *P*_*3*_, TSILA vs SSILA

All the *P* values with sigificantly stastistical difference were presented in bold

**Fig. 3:**
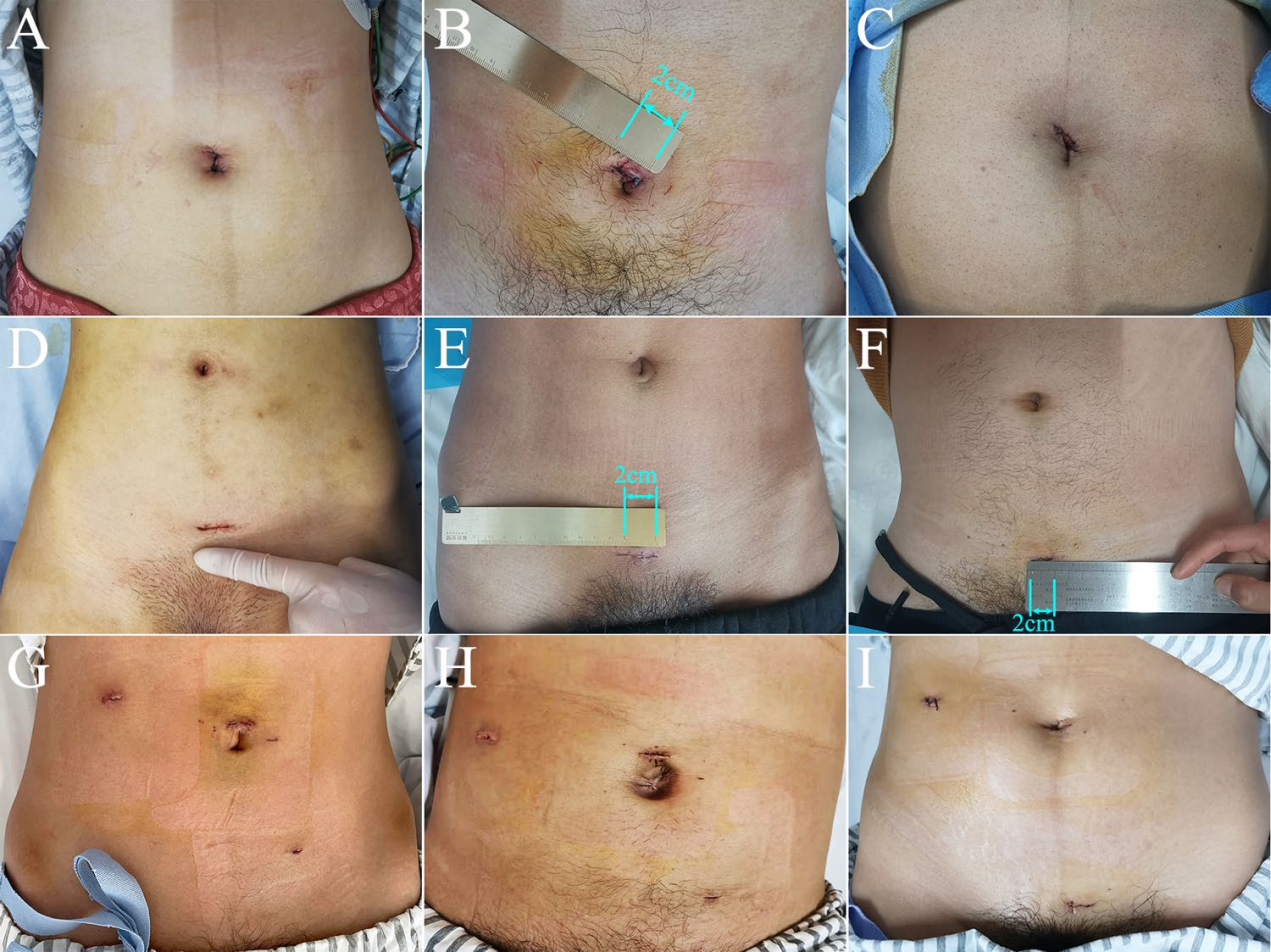
The length of each scar in the SILA group was about 2 cm, showing good cosmetic effect. **A, B,** and **C** A 2-cm incision around the umbilicus in the TSILA group. **D, E,** and **F** A 2-cm incision approximately 3 cm above the pubic symphysis in the SSILA group. **G, H,** and** I** Three incisions on the abdomen in the CLA group

Compared with CLA, TSILA was associated with significant reductions in postoperative pain within 6 h after the surgery, length of hospital stay, and hospital cost, while SSILA was associated with significant reductions in length of hospital stay, and hospital cost (Table 2). Significantly more patients in the two SILA groups were satisfied with the cosmetic results of the abdominal scar than those in the CLA group (Table 2, Fig. 3). However, compared with CLA, SSILA required significantly longer operative time (Table 2). Regarding the two SILA groups, the postoperative pain score in the SSILA group was significantly higher than that in the TSILA group (Table 2). Patients in the SSILA group had significantly higher satisfaction with the cosmetic results than those in the TSILA group (Table 2).

No severe postoperative complications occurred, including acute bleeding, abdominal abscess, incisional hernia, and conversion to open appendectomy. Mild incisional or intraabdominal infections with postoperative low fevers were noticed in 2 (3.0%) patients in the CLA group, 3 (5.1%) in the TSILA group, and 3 (6.3%) in the SSILA group (*P* = 0.69). Mild incisional infections were well managed with dressing changes and infrared therapy. Intraabdominal infections were well managed with antibiotics.

## Discussion

Whether single-port instruments are used or not, the laparoscope is always parallel to the other laparoscopic instruments. Thus, the “operating triangle” is relatively difficult to establish. Additionally, the “chopstick effect” and a “tubular field of vision” are inevitable, which results in a blind area of vision during the operation. This may increase the risk of complications, the learning curve for surgeons, and operative time, especially in the SSILA group. Our practice confirmed that skilled CLA techniques and familiarity with the vision of single-incision operations could gradually expand the surgical indications and shorten operative time after performing more than 10 cases of SILA. Surgeons can obtain stable surgical techniques for SILA after completion of 30 cases of TSILA or SSILA. This is consistent with previous reports [21]. Overall, the SILA technique presented in our study was more technically demanding than CLA.

Various studies have investigated the clinical outcomes of SILA compared to CLA. However, there are numerous debates regarding SILA [4, 8]. A previous meta-analysis and a clinical trial reported that SILA with single-port instruments is a safe and feasible procedure compared to CLA [22, 23]. Namely, SILA has faster recovery, better cosmetic effects, less postoperative pain, and lower conversion rates. Similar results were observed in the present study. Patients in the TSILA group and SSILA group were more cosmetically satisfied than those in the CLA group. However, SILA may lead to more postoperative pain at first day and longer operative time without improvements in short-term recovery [24]. Similarly, compared with CLA, SSILA was indeed associated with a significantly longer operative time and more postoperative pain. These results were due to the sophisticated entry and closure procedures near the abdominal wall. Some surgeons have suggested that the direct fascial puncture technique is not suitable to complete operations with instrument drag and may increase the risk of hernia [16]. However, it has been proven to be feasible and safe [16, 17], which is consistent with our results. Notably, regarding SSILA in this study, the 2-cm incision should also be closed in a layer-by-layer fashion under direct vision to prevent incisional hernia.

Even if SILA is performed with single-port instruments, long-term complications such as chronic pain, incisional hernia, and cosmetic results are still controversial [12, 13, 24, 25]. Those long-term complications certainly have significant burdens on the feasibility of these techniques. Follow-up of the patients who had undergone TSILA or SSILA at our hospital during the past 3 years found no severe chronic pain, obvious incisional hernia, or other severe complications. Despite the ongoing debate on single-incision laparoscopic surgery, it has made significant progress over the past few decades.

In our study, SILA was associated with less physical and mental stress of the patients, faster postoperative recovery, significantly higher cosmetic satisfaction, and significantly less hospital stay and cost, consistent with previous studies [5, 11, 16]. Although operative time was significantly shorter in the CLA group, it seems to have no significant effect on hospital stay and hospital cost. In other words, shorter operating time does not represent a significant cost saving. Therefore, SILA is recommended for the treatment of acute appendicitis.

There are some differences between TSILA and SSILA. First, TSILA is less considered for patients with a history of lower abdominal surgery, while this and cystostomy are absolute contraindications for SSILA. Second, for TSILA, a 2-cm arc incision around the umbilicus is hidden in the skin folds around the umbilicus. Although TSILA is more cosmetically appealing, it still damages the umbilicus, especially if the incision becomes infected. However, for SSILA, the incision is created approximately 3 cm above the pubic symphysis, without damaging the umbilicus. Additionally, the wound position is low and can be hidden in clothes. This also brings beneficial cosmetic effects. Third, SSILA was associated with significantly higher postoperative pain compared to TSILA. This might be attributed to the natural scar of the umbilicus, which has less nerve distribution and a simple tissue structure. Lastly, patients in the SSILA group were more cosmetically satisfied, which could be associated with the more hidden position of the scars.

These patients with appendicitis included in this study completed the lower abdominal computed tomography before the operation to exclude other abdominal diseases that are difficult to detect on physical examination. Then, the pathologic condition and position of the appendix can be roughly determined. Patients complicated with periappendiceal abscess are treated conservatively. Patients with severe appendicitis such as difficulty in exposure, gangrenes, or perforations of the appendix base, directly undergo CLA. According to our clinical experience, if a patient has the above complicated appendicitis, and a height < 1.5 m or a BMI > 28, the operating time of SILA easily exceeds 2 h. Moreover, most SILA procedures in these patients were ultimately converted to two-hole or three-hole laparoscopic appendectomy. This may increase the risk of complications. Therefore, in recent 3 years, these patients directly underwent CLA and were not included in our study.

It was undeniable that there were some complications, such as chronic pain, incisional infection, and postoperative fever in the two SILA groups. However, there was no significant difference between SILA and CLA. Therefore, TSILA and SSILA are safe and feasible surgical procedures for patients if they meet the inclusion and exclusion criteria in our study. At least surgeons could offer another option for patients with acute or chronic appendicitis when they request cosmetic incision and faster postoperative recovery.

Notably, the small sample size might have affected the accuracy of our results, which may result in certain bias influencing these. Therefore, future studies for SILA described in this study are needed.

## Conclusion

Our study demonstrated the feasibility of SILA with only conventional laparoscopic instruments. Compared with CLA, SILA was associated with better postoperative recovery and higher patient satisfaction with the cosmetic results.
